# Application of bacteriophage φPaP11-13 attenuates rat *Cutibacterium acnes* infection lesions by promoting keratinocytes apoptosis via inhibiting PI3K/Akt pathway

**DOI:** 10.1128/spectrum.02838-23

**Published:** 2024-01-10

**Authors:** Yuanyuan Liu, Ni Zhen, Danxi Liao, Jiahui Niu, Ruolan Liu, Zijiao Li, Zeyuan Lei, Zichen Yang

**Affiliations:** 1 Department of Plastic and Cosmetic Surgery, Xinqiao Hospital, Army Medical University (the Third Military Medical University), Chongqing, China; 2 Cadet Brigade 4, College of Basic Medicine, Army Medical University (the Third Military Medical University), Chongqing, China; 3 Institute of Burn Research, State Key Laboratory of Trauma, Burn and Combined Injury, Southwest Hospital Third Military Medical University, Chongqing, China; Tainan Hospital, Ministry of Health and Welfare, Tainan, Taiwan

**Keywords:** *Cutibacterium acnes*, acne vulgaris, phage, apoptosis, PI3K/Akt, *Propionibacterium acnes*

## Abstract

Acne vulgaris caused by antibiotic-resistant *Cutibacterium acnes* (*C. acnes*) infection is difficult to treat conventionally. Phages have been suggested as a potential solution, but research on the mechanism of phage treatment is inadequate. This research investigates the underlying molecular mechanisms of phage φPaP11-13 attenuating *C. acnes*-induced inflammation in rat models. We found that rats infected with *C. acnes* had higher average ear thickness, greater enrichment of inflammatory cells as shown by hematoxylin–eosin (HE) staining, and fewer TUNEL (TdT-mediated dUTP Nick-End Labeling)-positive keratinocytes visualized by IF staining. Moreover, an increase of IGF-1 and IGF-1 receptor (IGF-1r) was detected using the immunohistochemical (IHC) staining method, Western blot (WB), and quantitative real-time PCR (qRT-PCR) when infected with *C. acnes*, which was decreased after the application of phage φPaP11-13. By applying the IGF-1 antibody, it was demonstrated that the severity of *C. acnes*-induced inflammation was relevant to the expression of IGF-1. Through WB and qRT-PCR, activation of the PI3K/Akt pathway and a down-regulation of the BAD-mediated apoptosis pathway were discovered after *C. acnes* infection. Subsequently, it was shown that the activation of the PI3K/Akt pathway against BAD-mediated apoptosis pathway was alleviated after applying phage φPaP11-13. Furthermore, applying the IGF-1r inhibitor, Pan-PI3K inhibitor, and Akt inhibitor reversed the changing trends of BAD induced by *C. acnes* and phage φPaP11-13. This study demonstrates that one of the critical mechanisms underlying the attenuation of acne vulgaris by phage φPaP11-13 is lysing *C. acnes* and regulating keratinocyte apoptosis via the PI3K/Akt signaling pathway.

**IMPORTANCE:**

*Cutibacterium acnes* infection-induced acne vulgaris may cause severe physical and psychological prognosis. However, the overuse of antibiotics develops drug resistance, bringing challenges in treating *Cutibacterium acnes*. Bacteriophages are currently proven effective in MDR (multiple drug-resistant) *Cutibacterium acnes*, but there is a significant lack of understanding of phage therapy. This study demonstrated a novel way of curing acne vulgaris by using phages through promoting cell death of excessive keratinocytes in acne lesions by lysing *Cutibacterium acnes*. However, the regulation of this cell cycle has not been proven to be directly mediated by phages. The hint of ternary relation among "phage–bacteria–host" inspires huge interest in future phage therapy studies.

## INTRODUCTION

Acne vulgaris is among the most common inflammatory skin diseases worldwide, affecting 9.4% of the global population (1). The etiology of acne vulgaris is universally acknowledged to include elevated levels of skin sebum (2, 3), hyperkeratosis of the sebaceous gland (4), changes in differentiation of keratinocytes (5), infection by *Cutibacterium acnes (C. acnes*), and resultant immune cascades (6). Although acne is commonly seen and non-fatal, inappropriate treatment can lead to severe psychological and social problems (7), including but not limited to anxiety, depression (8), and even higher suicidal tendencies (9).

*Cutibacterium acnes* (*C. acnes*), also known as *Propionibacterium acnes* (10), is considered one of the significant contributors to the onset of acne vulgaris (11). Antibiotic-resistant *C. acnes* infection may result in failure of acne treatment, disruption of the skin microbiota, and widespread dissemination of antibiotic-resistant strains (12). To reduce antibiotic resistance, monotherapy of topical antibiotics is no longer recommended (13). Instead, according to the American Academy of Dermatology guidelines, topical retinoids and benzoyl peroxide are recommended in combination with antibiotics (14). However, with the widespread application of antibiotics for treating acne vulgaris, *C. acnes* resistance is becoming a more serious problem. Antibiotic-resistant *C. acnes* strains were discovered worldwide, primarily resistant to erythromycin and tetracycline (15). It was also demonstrated that *C. acnes* develops a more pronounced resistance to tetracyclines (16). Furthermore, in 2014, tetracycline-, clindamycin-, and erythromycin-resistant *C. acnes* strains were isolated from acne patients who had not accepted antibiotic therapies (17). In addition, the long-term use of tetracycline for acne treatment has been shown to cause specific side effects, which reduce the effectiveness of therapy by affecting patient adherence (18, 19). Non-antibiotic antimicrobial dressings were developed to reduce wound infection (20). However, with the increasing antibiotic resistance and multiple adverse effects, the demand for alternative therapies for acne vulgaris becomes more imperious.

Phages are viruses that naturally control bacterial populations by infecting and lysing bacteria with high specificity to a single species or strain of bacteria (21). Phage therapy, as an alternative to antibiotic therapies, is gathering renewed interest of researchers. Previous studies have shown that phages are highly efficient in treating antibiotic-resistant bacterial infections (22–25). It was reported that bacteriophage might have more effects in treating acne than antibiotics due to its amplification ability (21). In addition to alleviating inflammation, phages were reported to be able to modulate the gut metabolome by knocking down their targeted bacteria, providing more potential ways to treat infectious diseases (26). Phages had been applied in treating *Pseudomonas aeruginosa* infections in burn wounds and urinary tract infections in those undergoing transurethral prostatic resection, and phage treatment was non-inferior to antibiotic therapy (27, 28). *C. acnes* phage was first identified by Brzin in 1964 (29). *In vitro* experiments demonstrated the ability of *C. acnes* phage to control the growth of *C. acnes* by lysing *C. acnes* (30). Animal experiments and clinical trials have been performed to demonstrate the therapeutic effects of *C. acnes* phage in mouse models and acne patients (31, 32), revealing the significant potential of phages in treating antibiotic-resistant bacteria. However, the molecular mechanisms of phage treatment for antibiotic-resistant *C. acnes* infections are inadequately studied, making it highly detrimental to the understanding of phage therapy.

Our study aimed to verify the efficacy of phage and explore the specific molecular mechanisms in treatment of acne vulgaris by phage through rat models infected by antibiotic-resistant *C. acnes* strain Pacne11-13 and treated by phage φPaP11-13.

## MATERIALS AND METHODS

### Bacteria and phage preparation

The clinical *Cutibacterium acnes* strain Pacne11-13 was isolated from clinical samples of acne vulgaris patients and stored in the clinical lab of Xinqiao Hospital, Chongqing, China (33). The *C. acnes* strain was isolated, and the identification was conducted following the methods recommended by The Clinical and Laboratory Standards Institute ( CLSI (34). *C. acnes* Pacne11-13 was resuscitated and cultured in brain–heart infusion liquid mediums (BHI, pH 7.4, Oxoid, UK) at 37°C under anerobic conditions. The colony-forming unit (CFU) was measured through the plate count method (35). Bacteriophage φPaP11-13 was isolated from sewage collected in the medical wastewater treatment center in Xinqiao Hospital and reported previously (33). Phage φPaP11-13 against *C. acnes* was purified and identified with the double-layered agar method following the previous report (36). Bacteriophage titers (pfu/mL, plaque-forming units) were measured through the spot test described in the former study (36).Transmission electron microscopy (TEM, Hitachi HTT700, Japan) was used to observe and photograph phage morphology.

### Spectrophotometric assay

The optical density of the bacterial culture was measured at 600 nm wavelength (OD600) with a spectrophotometer (721N, Sunny Hengping Instrument, China) to monitor the growth of *C. acnes. C. acnes* Pacne11-13 was cultured in a BHI medium (pH 7.4, Oxoid, UK) at 37°C under anerobic conditions to the exponential phase (bacterial culture time = 30 hours, OD600 = 0.6890). Subsequently, with a sample size of 5, *C. acnes* cultured in flasks filled with 100 mL of the medium were respectively added with 10 µL phage (1 × 10^8^ pfu/mL) or 10 µL phosphate-buffered saline (PBS), co-cultured for 6 hours and measured with the spectrophotometer.

### Animal experiments

Approval for the animal experiments was obtained from the Medical Ethics Committee of the Second Affiliated Hospital (Xinqiao Hospital) of Army Medical University, PLA (approval number: AMUWEC2021580), and the animal experiments were conducted abiding by the accepted accord. Adult male Sprague–Dawley rats were purchased from the Experimental Animal Center at the Army Medical University. The acne rat models were established by injecting *C. acnes* (1.5 × 10^8^ CFU) into the ventral middle part of the rats' right ears (37). Once the acne models were established, with the sample size calculated following the previous study (38), the acne rats were equally divided into the *C. acnes* model group (***C. acnes***, *n* = 5), the antibiotics administration group (***C. acnes* +Antibiotics**, *n* = 5), and the phage administration group (***C. acnes* +Phage**, *n* = 5) through a randomized block design. The antibiotic administration group, the phage administration group, and the *C. acnes* model group were respectively locally injected with antibiotics (doxycycline and minocycline, 1:1, 0.40 mg in total), phage (3 × 10^11^ pfu), and the same volume of normal saline 6 hours after the models were established. Through a randomized block design, the control (**Control**, *n* = 5) group was set by injecting the same volume of normal saline, whereas the phage control group (**Phage**, *n* = 5) was set by locally injecting phage (3 × 10^11^ pfu) to confirm that no extra effects of phage were included. Exogenous IGF-1 protein (ab198570, Abcam) and IGF-1 antibody (ab63926, Abcam) were injected topically into the ear lesions of acne inflammation rat models to investigate the effect of IGF-1 on *C. acnes*-induced inflammation. All rat tissue samples were taken immediately after euthanasia of the rats.

### Hematoxylin–eosin (HE) staining

Sections of tissues from rats’ right ears were fixed with 4% formalin, embedded in paraffin, and sliced perpendicularly to the long axis. The 4-μm-thick slides were stained with hematoxylin–eosin (39), and the images were captured under an optical microscope (CX-21, OLYMPUS) for histopathological analysis. Data of ear thickness were measured by visualizing HE staining sections with pre-set graduated scales. Inflammatory cells were manually identified and counted using ImageJ software (ImageJ 1.53 k, National Institutes of Health, USA) under fields of 200 × 200 pixels randomly captured in each slide at 100 × magnification.

### Bacterial load quantification

About 5 mm × 5 mm full-thickness ear tissue specimens from rats’ right ears were weighed and homogenized in PBS. Then, the supernatant was collected and diluted in a series of concentration gradients from 10^−1^ to 10^−7^. Then, 0.01 mL dilutions of each gradient were evenly spread on BHI mediums and cultured at 37°C under anerobic conditions for 24 hours. The experiment was repeated three times for each sample. The bacterial load was expressed in CFU/g (CFU, colony-forming units).

### Immunohistochemical (IHC) staining

Immunohistochemistry was conducted on paraffin sections fixed in paraformaldehyde. Primary anti-IGF-1 antibody (1:100, ab9572, Abcam), primary anti-IGF-1r antibody (1:75, ab39675, Abcam), and secondary goat anti-rabbit antibody labeled with horseradish peroxidase (HRP, 1:200, AS-1107, Aspen) were used for immunohistochemistry. Briefly, paraffin sections were dewaxed and rehydrated. Antigen retrieval was performed, and the slides were cooled naturally, washed three times with PBS, and incubated in a 3% hydrogen peroxide solution for 10 minutes. The samples were then incubated with 5% BSA after natural drying and subsequently with the primary antibody at 4°C overnight and with HRP-labeled secondary antibody for 50 minutes at 37°C, followed by reaction with the freshly prepared DAB solution and counterstaining with hematoxylin. To score, three fields were randomly captured in each slide at 200 × magnification and scored using ImageJ software (ImageJ 1.53 k, National Institutes of Health, USA). The staining intensity scores of different areas in each field were categorized into 0 (negative), 1 (low-positive), 2 (positive), and 3 (high-positive) using the IHC Profiler plugin embedded in ImageJ software (40). The final IHC score of each field was expressed by adding staining intensity scores of four areas in each field weighted by percentages of the corresponding proportions.

### Immunofluorescence (IF) staining

IF staining was conducted on paraformaldehyde-fixed paraffin sections. Primary cytokeratin 10 antibody (1:200, 18343–1-AP, Proteintech Group) and secondary CY3-labeled goat anti-rabbit antibody (1:50, AS-1109, Aspen) were used for IF staining. TdT-mediated dUTP nick-end labeling (TUNEL) staining was performed with a TUNEL staining kit (*In Situ* Cell Death Detection Kit, Roche). In brief, paraffin sections were dewaxed, rehydrated, and washed three times with PBS. Antigen retrieval was performed by heating the slides in EDTA buffer in a microwave oven until boiling. After washing thrice with PBS, the samples were incubated with 3% hydrogen peroxide for 10 minutes. After being washed three times in PBS and drying, the samples were permeabilized with 0.5% Triton x-100 in PBS for 10 minutes and incubated with 5% BSA for 20 minutes. Subsequently, the samples were incubated with the primary antibody overnight at 4°C and washed thrice with PBS. The samples were then incubated with the secondary CY3-labeled antibody for 50 minutes at 37°C and washed thrice with PBS. The samples were incubated in TdT and dUTP mixed in a ratio of 1:9 for 60 minutes at 37°C, followed by washing thrice with PBS. The samples were counterstained with DAPI for 5 minutes and observed under a fluorescence microscope (BX63, OLYMPUS). The percentage of TUNEL-positive cells was evaluated using ImageJ software (ImageJ 1.53 k, National Institutes of Health, USA) under 100 × magnification.

### Cell culture

Rat keratinocytes (IMP-R143, IMMOCELL) were cultured in culture flasks containing medium (IMP-R143-1, IMMOCELL) with serum in a cell incubator at 37°C, 5% CO_2,_ and saturated humidity. The IGF-1 receptor inhibitor (IGF-1r inhibitor, Linsitinib), Pan-PI3K inhibitor (BKM120), and Akt inhibitor (AZD5363) were applied to determine the order of each signaling molecule in signaling pathways.

### Quantitative real-time PCR (qRT-PCR)

Total RNA was extracted from rats’ ear tissues using TRIzol reagent (Invitrogen) following the standard process (41). Reverse transcription was performed using the PrimeScriptRT reagent Kit with gDNA Eraser (TaKaRa) following the manufacturer’s instructions. Quantitative real-time PCR was performed using a qRT-PCR reagent kit (SYBR Premix Ex Taq, TaKaRa) on a StepOne Real-Time PCR System (Life Technologies). GAPDH was used as the endogenous normalization control, and relative expressions were calculated by the 2^-ΔΔCt^ method. Primer sequences for qRT-PCR were designed with the Primer-BLAST online tool, according to the design principles proposed in a previous study (42, 43). Original gene sequences were obtained from the NCBI gene database (44). The primers used for qRT-PCR were purchased from GeneCreate (Wuhan, China). Primer sequences are shown in Table 1.

**TABLE 1:** The primers for qRT-PCR

Names	Sequences (5’–3’)
GAPDH: forward	TGAAGGGTGGAGCCAAAAG
GAPDH: reverse	AGTCTTCTGGGTGGCAGTGAT
IGF1: forward	ACTTCAACAAGCCCACAGGC
IGF1: reverse	GACTTCTGAGTCTTGGGCATGTC
Bcl-2: forward	TCGTCGCTACCGTCGTGAC
Bcl-2: reverse	TCCCAGCCTCCGTTATCCT
Caspase-3: forward	GGAGAAATTCAAAGGACGGG
Caspase-3: reverse	GCATGGACACAATACACGGG
Caspase-9: forward	CATGATGTCTGTGTTCCAGGG
Caspase-9: reverse	TCTTGGCAGTCAGGTCGTTC
BAD: forward	GAGCAGGAAGACGCTAGTGCT
BAD: reverse	GGGTACGAACTGTGGCGACT

### Western blot analysis

In brief, rats’ ear tissues were dissected, homogenized on ice, and lysed with ice-cold lysis buffer. Total protein was extracted from the histiocyte lysate, separated by gel electrophoresis with SDS-PAGE, and transferred onto a PVDF membrane (LC2007, Invitrolon). The membranes were incubated with the following primary antibodies overnight at 4°C: anti-IGF-1 antibody (1:250, ab63926, Abcam), anti-IGF-1 receptor antibody (1:500, ab39398, Abcam), anti-PI3K antibody (1:200, ab278545, Abcam), anti-pan-Akt antibody (1:500, ab8805, Abcam), anti-Phospho-Akt (Thr308) antibody (1:500, ab38449, Abcam), anti-Phospho-Akt (Ser473) antibody (1:5000, ab81283, Abcam), anti-BAD antibody (1:1000, ab32445, Abcam), anti-NF-κB antibody (1:200, ab16502, Abcam), anti-MAPK antibody (1:1000, ab170099, Abcam), anti-MEK antibody (1:200, ab140372, Abcam), anti-Phospho-MEK antibody (1:500, ab194754, Abcam), anti-ERK antibody (1:10000, ab184699, Abcam), anti-Phospho-ERK antibody (1:1000, ab201015, Abcam), and anti-GAPDH antibody (1:2500, ab9485, Abcam). Subsequently, the membranes were incubated with secondary HRP-conjugated goat anti-rabbit antibody at room temperature for 1 hour and washed with TBST buffer. Protein bands were visualized using a chemiluminescence kit (E-IR-R304A, Elabscience). GAPDH served as the internal control. Determination of protein concentrations was conducted using ImageJ software (ImageJ 1.53 k, National Institutes of Health, USA).

### Statistical analysis

Data were expressed as mean ± SD from at least three independent replicates. Statistical analyses were conducted using SPSS software (SPSS 24.0, IBM), and a two-tailed Student *t*-test was adopted to analyze the differences between the two groups. Differences were considered statistically significant at *P* < 0.05.

## RESULTS

### Bacteriophage φPaP11-13 showed lytic activity against *C. acnes.*

**Fig 1:**
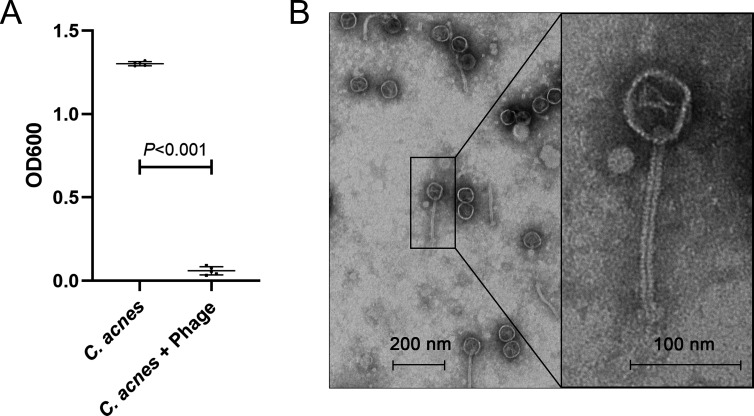
(**A**) The spectrophotometric assay for *C. acnes* growth with and without phage φPaP11-13 at 600 nm. (**B**) Representative electron micrographs showing phage φPaP11-13 virion morphology.

The OD600 value of *C. acnes* treated with phage φPaP11-13 for 6 hours was significantly lower than that of *C. acnes* with PBS (Fig. 1), indicating that phage φPaP11-13 was capable of lysing *C. acnes* effectively.

### The *C. acnes* + Phage group presented more apoptosis of keratinocytes and less inflammation in acne lesions than the *C. acnes* group

**Fig 2:**
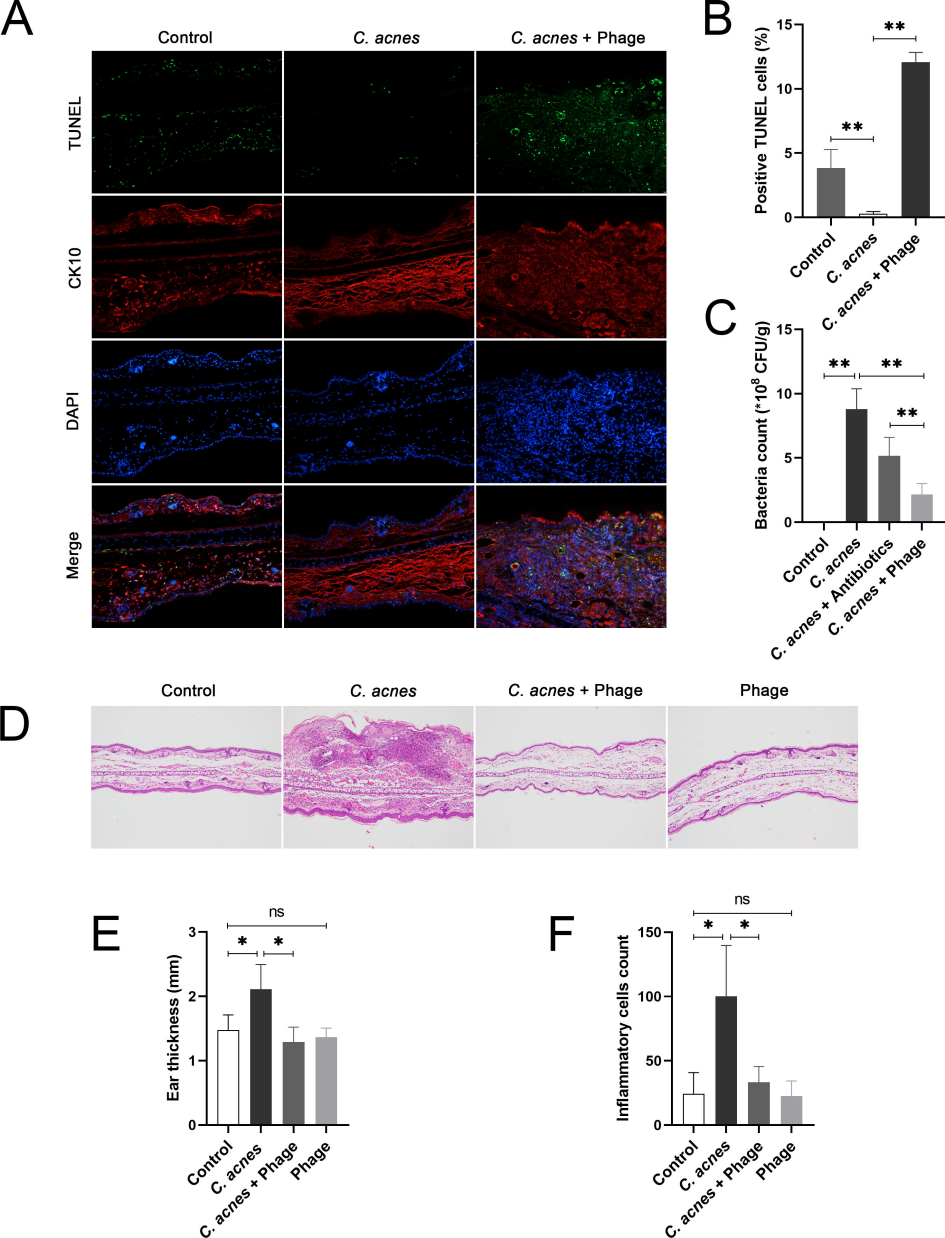
(**A**) Representative images of IF staining of rats’ ear tissues in the control group, the *C. acnes* group*,* and the *C. acnes* +Phage group at 200 × magnification. The TUNEL method was performed to detect cell apoptosis in tissues, and TUNEL-positive cells were stained green. CK10 was stained in red. The nuclei were stained with DAPI in blue. (**B**) Comparison of the percentage of TUNEL-positive cells among groups presented in IF staining images. (**C**) The bacterial count of tissues from rats’ ears in the control group, the *C. acnes* group, the *C. acnes* +antibiotics group, and the *C. acnes* +Phage group. (**D**) Representative images of HE staining of rats’ ear tissues from rats’ ears in the control group, the *C. acnes* group, the *C. acnes* +Phage group, and the phage group. (**E**) Measurements of the average thickness of rats’ ears in HE staining images. (**F**) Determination of the inflammatory cell count of tissues of rats’ ears in HE staining images. Ns, no significance; * *P* < 0.05; ** *P* < 0.01.

TUNEL staining was performed to detect cell apoptosis in rats’ ear tissues. The percentage of TUNEL-positive cells in tissues in the *C. acnes* group was significantly lower than that in the control group. However, the percentage of TUNEL-positive cells was significantly higher in the *C. acnes* +Phage group than in the *C. acnes* group (Fig. 2A and B). It is worth noting that compared with the control group, CK10 expression was slightly elevated in the *C. acnes* group, and the location of TUNEL-positive cells in the *C. acnes* +Phage group largely overlapped with that of the CK10-positive region, suggesting that the increased apoptosis in the *C. acnes* +Phage group primarily occurred in keratinocytes. Besides, phage significantly reduced bacterial load in tissues infected with *C. acnes* and produced a more pronounced effect in reducing bacterial load than antibiotics (Fig. 2C). HE staining was conducted to evaluate the severity of inflammation in rats’ ear tissues. HE staining showed a significant increase in average ear thickness and a significant local enrichment of inflammatory cells in the *C. acnes* group than in the control group, both of which indicated the development of inflammation after infection; however, both the increased average ear thickness and count of inflammatory cells induced by *C. acnes* were significantly reduced after the application of phage in the *C. acnes* +Phage group (Fig. 2D through F).

### *C. acnes* elevated the expression of IGF-1 and IGF-1r, while phage inhibited the up-regulation of IGF-1 and IGF-1r by lysing *C. acnes*

**Fig 3:**
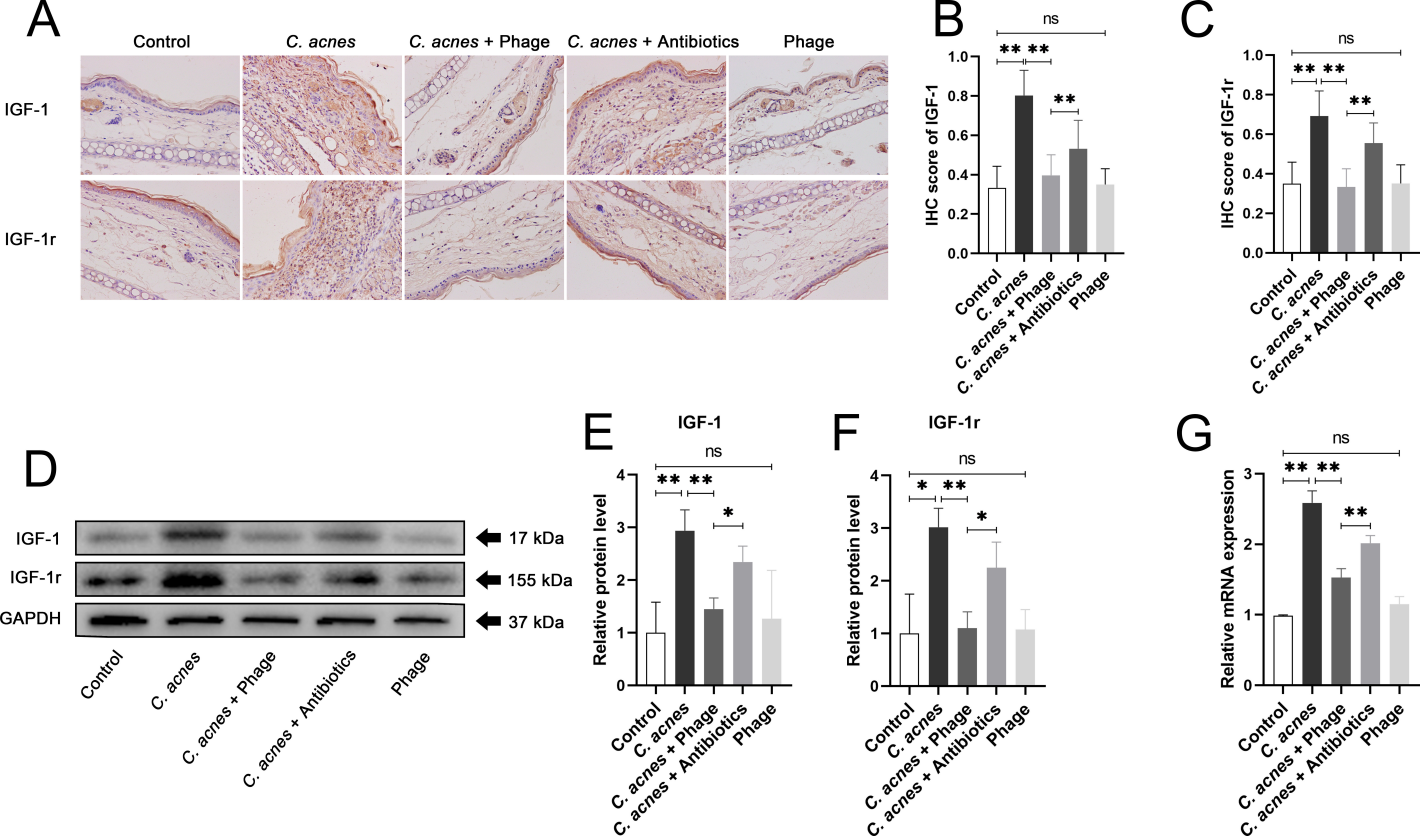
(**A**) Representative images of IHC staining of IGF-1 and IGF-1r in the control group, the *C. acnes* group, the *C. acnes* +Phage group, the *C. acnes* +Antibiotics group, and the phage group at 400 × magnification. (**B**) IHC scores of IGF-1 in IHC staining images (**C**) Measurement of IHC scores of IGF-1r in IHC staining images. (**D**) The protein bands of IGF-1, IGF-1r, and GAPDH in the control group, the *C. acnes* group, the *C. acnes* +Phage group, the *C. acnes* +Antibiotics group and the phage group are shown by the Western blot method. (**E**) The expression levels of IGF-1 protein were measured by the WB method. GAPDH served as the internal control. (**F**) The expression levels of IGF-1r protein were measured by the WB method. GAPDH served as the internal control. (**G**) Relative expression of mRNA of IGF-1 in the control group, the *C. acnes* group, the *C. acnes* +Phage group, the *C. acnes* +Antibiotics group, and the phage group detected by the qRT-PCR method. GAPDH served as the internal control. Ns, no significance; * *P* < 0.05; ** *P* < 0.01.

IHC staining was conducted to evaluate the expression level and expression location of IGF-1 and IGF-1r in rats’ ear tissues. IHC staining showed a pronounced up-regulation of IGF-1 and IGF-1r in the *C. acnes* group compared with that in the control group, as well as a remarkable down-regulation of IGF-1 and IGF-1r in the *C. acnes* +Phage group. Notably, IGF-1 and IGF-1r were primarily expressed in similar areas near the skin’s epidermis, indicating a close relationship between the expression of IGF-1 and that of IGF-1r (Fig. 3A). IHC staining was analyzed using ImageJ software to examine the relative expression of IGF-1 and IGF-1r. IGF-1 and IGF-1r were upregulated in the *C. acnes* group compared to the control group and significantly down-regulated in the *C. acnes* +Phage group. IGF-1 and IGF-1r expressions were significantly more down-regulated in the *C. acnes* +Phage group than in the *C. acnes* +antibiotics group (Fig. 3B and C). WB analysis was applied to detect the protein level of IGF-1 and IGF-1r. Similar to the results of IHC staining, the WB analysis showed that the overexpression of IGF-1 and IGF-1r protein induced by *C. acnes* was significantly reduced in the *C. acnes* +phage group compared with the *C. acnes* +Antibiotics group (Fig. 3D through F). QRT-PCR was performed to detect the relative mRNA expression of IGF-1 in rats’ ear tissues. It was shown by qRT-PCR that the relative expression of IGF-1 was significantly lower in the *C. acnes* +Phage group than in the *C. acnes* +antibiotics group (Fig. 3G), which was in accordance with the results shown in the WB analysis.

### There was a significant positive correlation between the concentration levels of IGF-1 protein and the severity of inflammation caused by *C. acnes*

**Fig 4:**
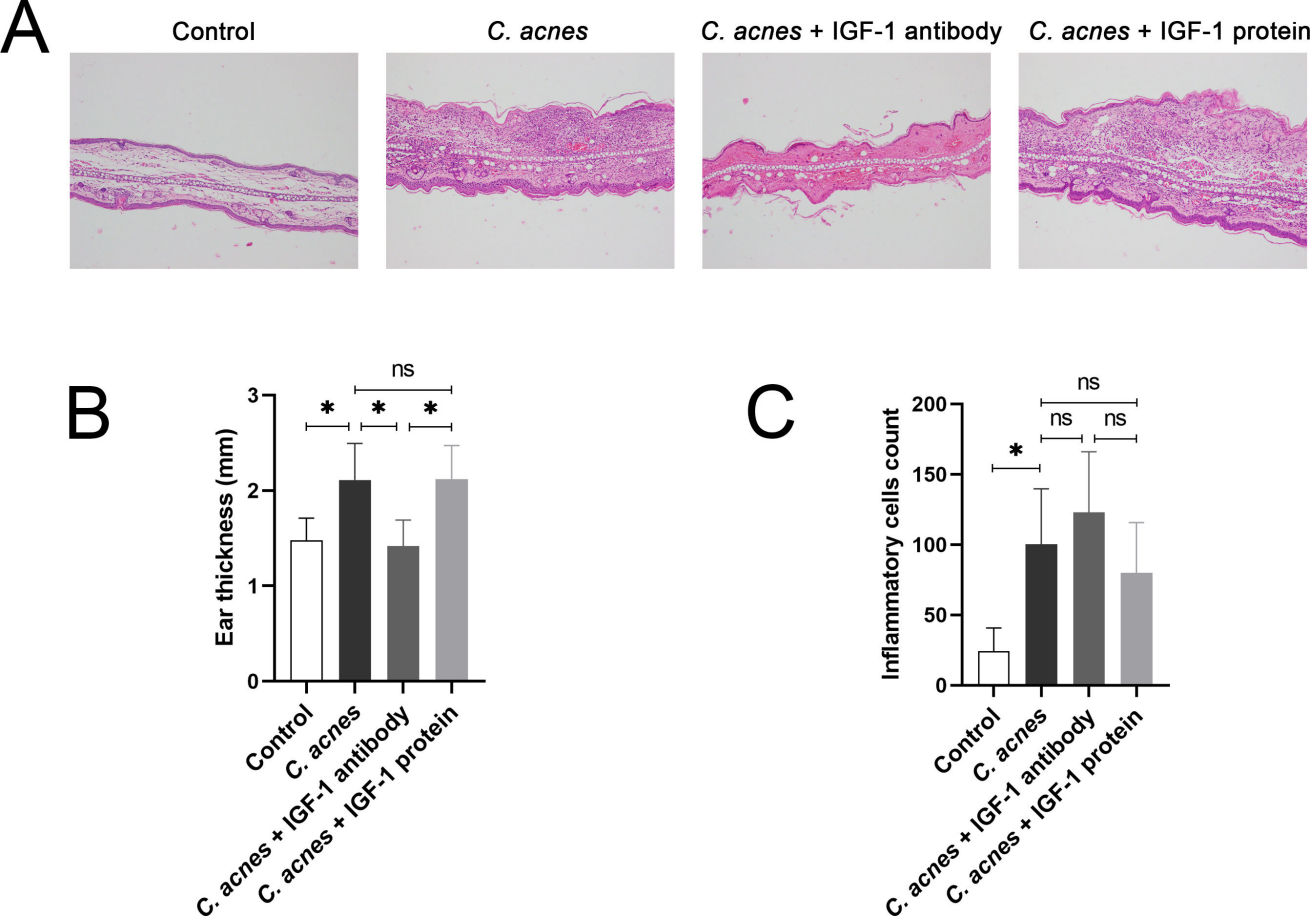
(**A**) Representative images of HE staining of ear tissues from the control group, the *C. acnes* group, the *C. acnes* +IGF-1 antibody group, and the *C. acnes* +IGF-1 protein group at 100 × magnification. (**B**) Measurements of the average thickness of rats’ ears in the control group, the *C. acnes* group, the *C. acnes* +IGF-1 antibody group, and the *C. acnes* +IGF-1 protein group. (**C**) Determination of inflammatory cell count of tissues of rats’ ears in the control group, the *C. acnes* group, the *C. acnes* +IGF-1 antibody group, and the *C. acnes* +IGF-1 protein group. Ns, no significance; * *P* < 0.05; ** *P* < 0.01.

Artificially added IGF-1 antibody and IGF-1 protein were applied to established rat inflammatory models infected with *C. acnes* to explore the effect of IGF-1 on the severity of inflammation caused by *C. acnes*. It was shown by HE staining that after the infection of *C. acnes*, the application of IGF-1 protein significantly increased the average thickness of rats’ ears compared to the application of IGF-1 antibody, while the number of inflammatory cells exhibited no noticeable difference (Fig. 4A through C).

### As a result of the downregulation of the PI3K/Akt pathway, BAD proteins and downstream mitochondria apoptotic pathway were upregulated in the *C. acnes* +Phage group compared with the *C. acnes* group

**Fig 5:**
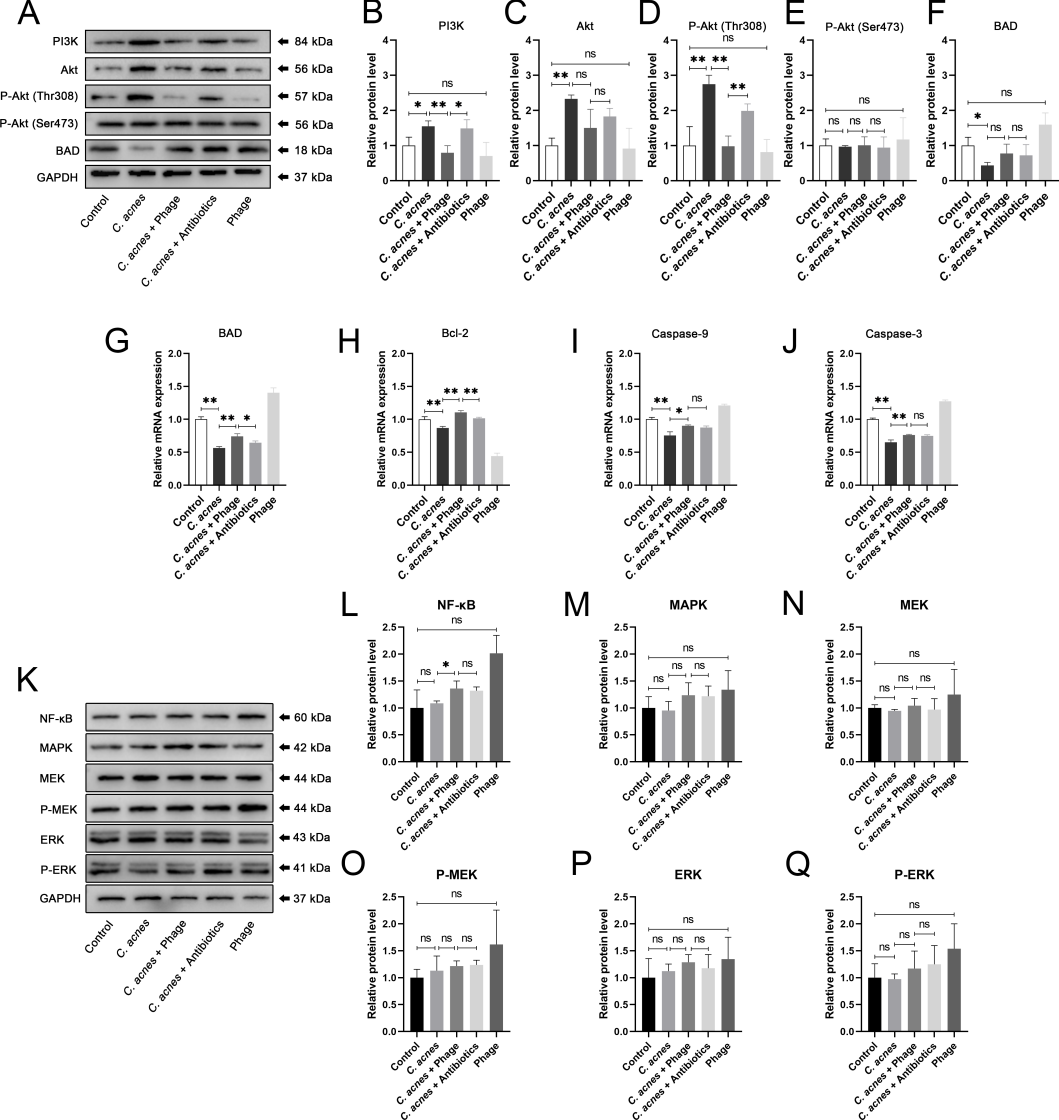
(**A**) The protein bands of PI3K, Akt, Phospho-Akt (Thr308), Phospho-Akt (Ser473), BAD, and GAPDH in the control group, the *C. acnes* group, the *C. acnes* +Phage group, the *C. acnes* +Antibiotics group and the phage group showed by the Western blot method. (**B**)-(**F**) The expression levels of PI3K, Akt, Phospho-Akt (Thr308), Phospho-Akt (Ser473), and BAD proteins measured by the WB method. GAPDH served as the internal control. (**G**)-(**H**) Relative expression of the mRNA of BAD, Bcl-2, caspase-9, and caspase-3 in the control group, the *C. acnes* group, the *C. acnes* +Phage group, the *C. acnes* +Antibiotics group, and the phage group detected by the qRT-PCR method. GAPDH served as the internal control. (**K**) The protein bands of NF-κB, MAPK, MEK, Phospho-MEK, ERK, Phospho-ERK, and GAPDH in the control group, the *C. acnes* group, the *C. acnes* +Phage group, the *C. acnes* +Antibiotics group, and the phage group showed by the Western blot method. (**L**)-(**Q**) The expression levels of NF-κB, MAPK, MEK, Phospho-MEK, ERK, and Phospho-ERK proteins measured by the WB method. GAPDH served as the internal control. Ns, no significance; * *P* < 0.05; ** *P* < 0.01.

WB analysis was performed to detect the expression level of PI3K/Akt relevant proteins (Fig. 5A). The expression level of PI3K was significantly elevated in the *C. acnes* group than in the control group. Subsequently, after the application of phage in the *C. acnes* + Phage group, the activation of PI3K was significantly down-regulated compared to that in the *C. acnes* group, which was substantially more pronounced than that in the *C. acnes* + Antibiotics group (Fig. 5B). As a classical protein downstream of PI3K, Akt expression was correspondingly upregulated in the *C. acnes* group compared to that in the control group. However, no statistically significant change of Akt expression was shown in the *C. acnes* + Phage group compared to that in the *C. acnes* group, but a declining trend was observed (Fig. 5C). Nonetheless, the expression level of P-Akt (Thr308) showed a similar expression trend to PI3K protein, with no significant change in the expression level of P-Akt (Ser473) (Fig. 5D and E), indicating that the activation of Akt induced by *C. acnes* in this signaling pathway was primarily regulated via the Thr308 phosphorylation site instead of Ser473 in Akt protein. These results suggested that the PI3K/Akt signaling pathway was activated in *C. acnes*-infected tissues, and this activation was inhibited in the *C. acnes* + Phage group. Moreover, phage exhibited higher activity against *C. acnes* than antibiotics; therefore, the activation of the PI3K/Akt signaling pathway induced by *C. acnes* was lower in the group treated with phage φPaP11-13 compared to that treated with antibiotics.

As an apoptosis-related protein regulated by the PI3K/Akt signaling pathway, the expression level of BAD protein was significantly down-regulated in the *C. acnes* group compared with that in the control group. Subsequently, the BAD protein expression level showed some up-regulation trend in the *C. acnes* + Phage and the *C. acnes* + Antibiotic groups, but there was no statistical difference (Fig. 5F). QRT-PCR was conducted to evaluate the relative mRNA expression of BAD, Bcl-2, caspase-9, and caspase-3. Consistent with the results of WB analysis, the relative mRNA expression of BAD was significantly decreased in the *C. acnes* group compared with that in the control group. After phage application in the *C. acnes* + Phage group, the relative mRNA expression of BAD was significantly upregulated, which was significantly higher than that in the *C. acnes* + Antibiotics group (Fig. 5G). Respectively, at transcriptional and translational levels, the results of qRT-PCR and WB analysis on the expression of BAD together indicated that phage φPaP11-13 suppressed the *C. acnes*-induced decline in BAD protein expression by lysing *C. acnes*.

The relative mRNA expression of Bcl-2 was statistically significantly but slightly down-regulated in the *C. acnes* group and upregulated in the *C. acnes* + Phage group and the *C. acnes* + Antibiotics group (Fig. 5H), indicating that the expression of Bcl-2 may be upregulated at the transcriptional level by negative feedback after a decrease in activity induced by binding with BAD protein, which was upregulated in the *C. acnes* + Phage group. The relative mRNA expression of caspase-9 and caspase-3 showed similar trends, in which both were significantly down-regulated in the *C. acnes* group compared to the control group and were significantly upregulated in the *C. acnes* + Phage group and the *C. acnes* + Antibiotics group compared to the *C. acnes* group, with no statistical difference in expression between the *C. acnes* + Phage and the *C. acnes* + Antibiotic groups (Fig. 5I and J). The results of the relative mRNA expression of caspase-9 and caspase-3 suggested that phage φPaP11-13 can inhibit *C. acnes*-induced down-regulation of apoptosis by lysing *C. acnes*. The relative mRNA expression of NF-κB, MAPK, MEK, Phospho-MEK, ERK, and Phospho-ERK exhibited no apparent trends (Fig. 5K through 5Q), which indicated that these signal factors were not remarkably regulated in the infection of *C. acnes*.

### IGF-1r inhibitor, Pan-PI3K inhibitor, and Akt inhibitor attenuated IGF-1-induced hypo-expression of BAD through the PI3K/Akt signaling pathway

**Fig 6:**
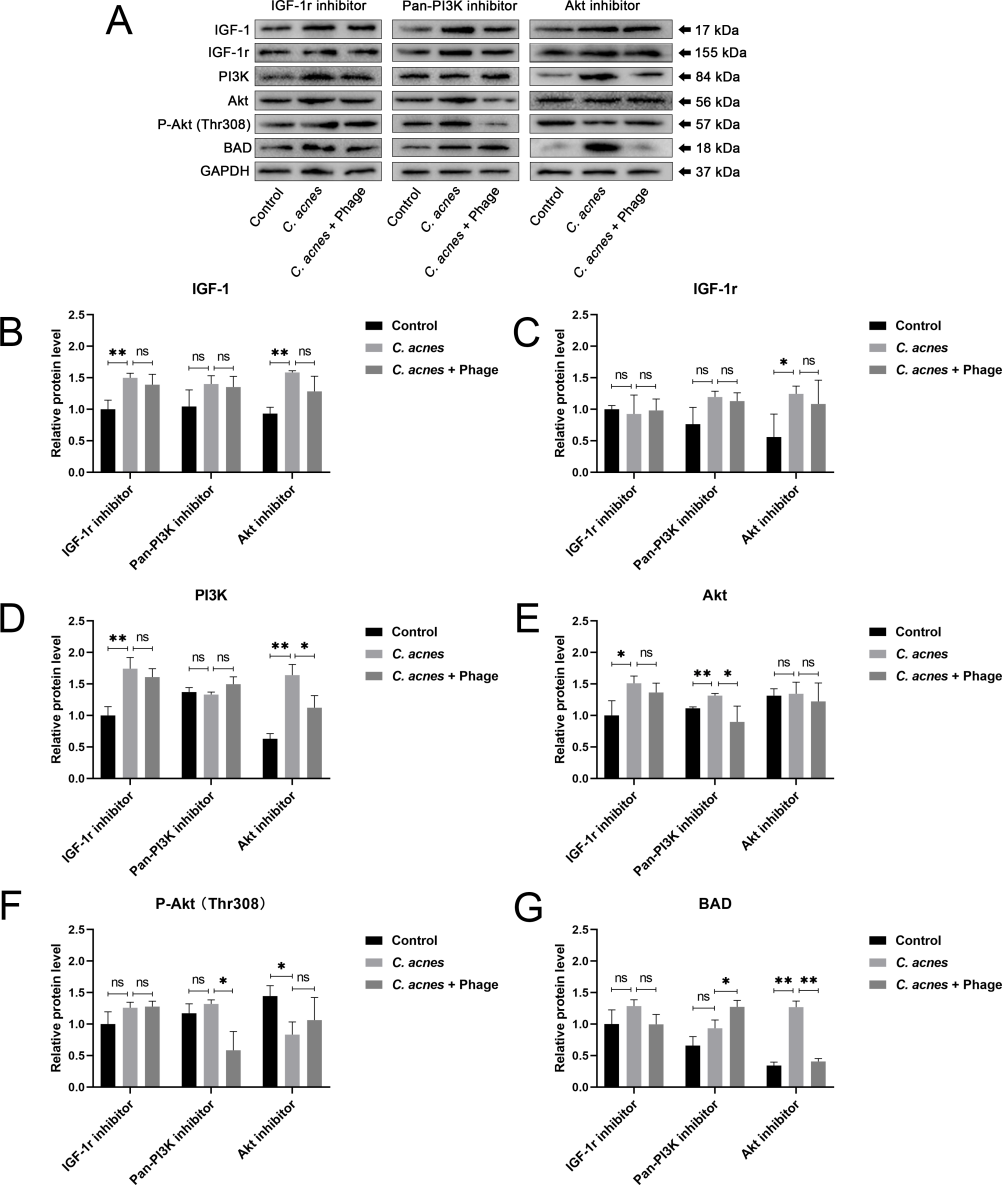
(**A**) The protein bands of IGF-1, IGF-1r, PI3K, Akt, Phospho-Akt (Thr308), BAD, and GAPDH in the control group, *C. acnes* group and *C. acnes* + Phage group shown by the Western blot method. (**B**)-(**G**) The expression levels of IGF-1, IGF-1r, PI3K, Akt, Phospho-Akt (Thr308), and BAD proteins measured by the WB method. GAPDH served as the internal control. Ns, no significance; * *P* < 0.05; ** *P* < 0.01.

WB analysis was performed to detect the expression level of PI3K/Akt relevant proteins after applying the IGF-1r inhibitor, Pan-PI3K inhibitor, and Akt inhibitor (Fig. 6A). In contrast to the remarkable upregulation of Akt expression after infection with *C. acnes* shown in Fig. 5C, the upregulation of Akt in the *C. acnes* group was attenuated after applying the Pan-PI3K inhibitor (Fig. 6E). More apparently, the significant up-regulation of P-Akt (Thr308) shown in Fig. 5C was weakened after the application of the Pan-PI3K inhibitor, showing no statistical upregulation of P-Akt(Thr308) in the *C. acnes* group (Fig. 6F), suggesting that PI3K was in the upstream of Akt. It was shown that no significant change occurred to the expression of PI3K after applying the Akt inhibitor (Fig. 6D), further suggesting that Akt was downstream of PI3K in the signaling pathway. Neither Pan-PI3K inhibitor nor Akt inhibitor reversed the expression trends of IGF-1 and IGF-1r (Fig. 6B and C). The application of the IGF-1r inhibitor exhibited inhibition of the expression of P-Akt (Thr308) in the *C. acnes* group in comparison with the up-regulation of P-Akt (Thr308) in the *C. acnes* group without the IGF-1r inhibitor shown in Fig. 5D (Fig. 6F), indicating that IGF-1/IGF-1r protein was closely related with the activation of the PI3K/Akt signaling pathway. It was shown that all IGF-1r inhibitor, Pan-PI3K inhibitor, and Akt inhibitor reversed the down-regulation of BAD induced by *C. acnes* (Fig. 6G), suggesting that BAD was in the downstream of the IGF-1/IGF-1r protein and PI3K/Akt signaling pathway.

## DISCUSSION

Our research demonstrated that the bacteriophage φPaP11-13 attenuates *C. acnes* infection in rat acne models and upregulates keratinocyte apoptosis via the PI3K/Akt signaling pathway by lysing *C. acnes*. Animal experiments proved that phage application took a similar or even more potent effect than antibiotics in reducing inflammation and suppressing *C. acnes*-induced inhibition of keratinocyte apoptosis, revealing the prospective application value of phage as an alternative to antibiotics.

The pathogenesis of acne is known as the overgrowth of *C. acnes* in skin lesions and resultant inflammatory immune cascades, as well as changes in skin sebum and the sebaceous gland (5). Until now, the global burden of acne remains high (45). Conventional treatment for acne includes topical retinoid, benzoyl peroxide, topical antibiotics, and oral antibiotics (14). With the increasing problem of antibiotic resistance, anti-bacterial therapy for acne needs to be optimized (46). Bacteriophage’s ability to eliminate bacterial infection was first put forward in the 1910 s (47). After a century of research, phage is now considered a potential alternative to antibiotics (48). However, the precise mechanism of phage treatment for acne vulgaris remains unclear. To explore the underlying molecular mechanisms of phage attenuating *C. acnes*-induced inflammation, we established rat inflammatory models and conducted subsequent experiments.

*C. acnes* phage was described previously to have similarly sized heads with 50 nm diameter and 150 nm long and flexible tails and was shown to have broad ability to kill clinical isolates of *C. acnes* (49). *In vitro* experiments demonstrated that *C. acnes* phage could lyse *C. acnes* (30). Likewise, we observed phage’s lysing ability against *C. acnes* through the spectrophotometric assay. Moreover, previous animal experiments showed that *C. acnes* phage could reduce the size of acne inflammatory lesions on mice’s backs (31) as well as the bacterial load and inflammation in acne lesions (50). Correspondingly, we observed a reduction of the thickness and inflammatory cells in rats’ ears after applying phage φPaP11-13 to acne lesions in rats’ ears infected by *C. acnes*. Our study and previous studies together demonstrated that phages could reduce inflammation by lysing bacteria and reducing the bacterial load of acne lesions.

Topical growth factors were reported to have a profound association with skin healing (51). Previous studies had demonstrated that IGF-1 was elevated in acne patients (52, 53), indicating that IGF-1 and downstream signaling pathways were associated with the severity of acne. Consistent with previous studies, we observed an increase of IGF-1 and IGF-1r in rat acne models and a strong correlation between the concentration of IGF-1 and the severity of inflammation, and phage was able to reduce *C. acnes*-induced over-expression of IGF-1 by lysing *C. acnes*.

As mentioned before, the etiology of acne vulgaris is also closely related to hyperkeratosis (4), which is caused by the hypo-apoptosis of keratinocytes. A previous study has proven that the proliferation of keratinocytes was enhanced by IGF-1 via the activation of IGF-1r in the development of acne vulgaris (54). Correspondingly, our experiments showed that keratinocyte apoptosis was significantly down-regulated by *C. acnes* and that phage φPaP11-13 was capable of restoring the hypo-apoptosis induced by *C. acnes,* suggesting that the application of phage could influence keratinocyte apoptosis through lysing *C. acnes* to attenuate *C. acnes*-induced inflammation. However, the specific mechanisms were not reported before. As reported previously, the dysregulation of the PI3K/Akt pathway was relevant to acne vulgaris (55), which is consistent with our observation that the expression of PI3K and Akt was significantly up-regulated after the infection of *C. acnes.* We also found a pronounced reduction of the expression of BAD and its downstream apoptosis-related proteins: caspase-9 and caspase-3, after the infection of *C. acnes*. Furthermore, after the infection of *C. acnes*, the application of the Pan-PI3K inhibitor inhibited the up-regulation of Akt and P-Akt (Thr308), whereas the Akt inhibitor had no significant impact on PI3K, indicating that PI3K was in the upstream of Akt. Neither the Pan-PI3K inhibitor nor the Akt inhibitor reversed the upregulation of IGF-1, but the application of the IGF-1r inhibitor significantly inhibited the activation of P-Akt (Thr308), revealing that IGF-1 was in the upstream of the PI3K/Akt pathway. All of the IGF-1r inhibitors, Pan-PI3K inhibitors, and Akt inhibitors reversed the down-regulation of BAD induced by *C. acnes*, suggesting that BAD was downstream of the IGF-1 and PI3K/Akt signaling pathway. In summary, it was demonstrated that *C. acnes* reduced keratinocyte apoptosis by down-regulating BAD protein and downstream apoptosis-relevant proteins by regulating the IGF-1/PI3K/Akt signaling pathway. However, phage φPaP11-13 inhibited *C. acnes*-induced over-activation of the IGF-1/PI3K/Akt signaling pathway and hypo-apoptosis of keratinocytes by lysing *C. acnes*.

In conclusion, our study aimed to investigate the therapeutic effect of phage application in rat inflammatory models infected with *C. acnes* and its underlying molecular mechanisms of alleviating inflammation. With *in vitro* experiments and animal experiments, we demonstrated that the infected lesions of *C. acnes* in a rat inflammatory model were significantly attenuated by phage and that phage had a modulatory effect in keratinocyte apoptosis by lysing *C. acnes* to reduce the over-proliferation of keratinocytes in rats’ ear tissues, revealing the great potential of phage as an alternative therapy to antibiotics. However, phage therapy also has potential risks that cannot be ignored and require further research. For example, the optimal therapeutic dose, administration time, treatment frequency, duration, evaluation of efficacy, and other aspects that are close to clinical practice have not been well-studied (56). In addition, phage capsid proteins may elicit unknown immune responses as an antigen (57), posing a risk to the therapy. It was discovered that bacteria have developed resistance to phages as they have already developed to antibiotics (58, 59), through bacteria evolution (60). The aforementioned discovery shows that although our experiments illustrate that phage φPaP11-13 has some effect on treating *C. acnes* infection, it cannot be applied to actual clinical therapy for the time being. There is still room for further research on phage treatment of *C. acnes* infection, which is also our next research direction.

## Data Availability

The data sets used and/or analyzed during the current study are available from the corresponding author upon reasonable request.
